# Effects of Climate Warming on the Performance of *Gynaephora alpherakii* (Lepidoptera: Lymantriidae) Larvae in a Tibetan Alpine Meadow

**DOI:** 10.1002/ece3.70978

**Published:** 2025-02-09

**Authors:** Rui Cao, Han Chen, Kezhi Zheng, Dajie Nong, Menglei Jiang, Ziyu Zhang, Xinwei Wu, Peng Xie

**Affiliations:** ^1^ Jiangsu Key Laboratory for Eco‐Agricultural Biotechnology Around Hongze Lake, Collaborative Innovation Center of Regional Modern Agriculture & Environmental Protection Huaiyin Normal University Huaian China; ^2^ Department of Ecology, School of Life Sciences Nanjing University Nanjing China

**Keywords:** body size, feeding activity, grassland caterpillar, invertebrate, Qinghai–Tibet plateau

## Abstract

The performance of invertebrate herbivores in grasslands can be influenced by climate warming, but there is a lack of experimental evidence, particularly in high elevation areas. We conducted two complementary experiments to investigate the effect of experimental warming on the performance of the grassland caterpillar *Gynaephora alpherakii*, a notorious pest species in the alpine Tibetan meadow. The first field experiment examined the effect of warming (nonwarmed vs. warmed) on the feeding behavior, growth, and development rate of the caterpillars. The second chamber experiment explored the relationship between temperature and caterpillar appetite, excrement mass, respiration rate, or change of caterpillar weight. Results show that warming significantly decreased fresh body mass of caterpillars by 27.5%, cocoon volume by 61.1%, and egg production per female moth by 26.9% at the end of the field experiment. Warming did not affect cocooning time but significantly increased feeding time of caterpillars during the field experimental period. The independent chamber experiment revealed a significant and positive correlation between caterpillar appetite, excrement mass, respiration rate, and temperature. However, except the first examination, there was a significant negative correlation between changes in caterpillar weight and temperature. Stepwise regression analysis indicated that excrement mass had the greatest influence on caterpillar weight. The weight loss of caterpillars to warming might thus be attributed to elevated metabolic rates at higher temperatures, and the behavioral adaptations failed to compensate for the physiological‐induced weight loss. These findings suggest that climate warming can modify the performance and thus the fitness of invertebrate herbivores in high elevation areas.

## Graphical abstract

Warming significantly increased caterpillar feeding time. Warming significantly decreased caterpillar body size. The behavioral adaptations failed to compensate for the physiological‐induced weight loss.

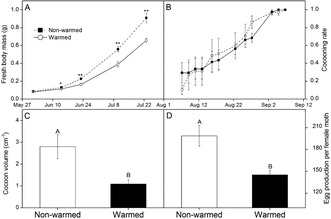

## 1 Introduction

Climate change poses a significant threat to ecosystems globally, with rising temperatures profoundly affecting biological processes and interactions (IPCC 2021; Harvey et al. 2023). Among the most vulnerable are invertebrate herbivores, which play essential roles in maintaining plant community structure, nutrient cycling, and energy flow within grassland ecosystems (Hulme 1994; Kempel et al. 2015; McCary and Schmitz 2021). Understanding how climate warming impacts these organisms is critical for predicting changes in community dynamics and ecosystem functioning.

A well‐documented response to warming in ectotherms is a reduction in body size, governed by the temperature‐size rule, which posits an inverse relationship between temperature and adult body size (Angilletta et al. 2004; Kingsolver and Huey 2008). Faster growth and developmental rates under elevated temperatures often result in smaller adult sizes, aligning with the intraspecific version of Bergmann's rule (Blackburn et al. 1999). While this phenomenon is commonly observed, exceptions exist, especially in high‐elevation or extreme environments, where body size responses may deviate due to unique ecological and physiological adaptations (Zhao et al. 2014; Xi et al. 2016). These complexities highlight the need for targeted studies to elucidate the mechanisms driving body size changes under warming, particularly in understudied taxa like high‐elevation invertebrates.

Alpine regions, such as the Tibetan Plateau, are experiencing rapid warming and altered precipitation patterns, making them critical areas for studying climate impacts on biodiversity (Wu et al. 2011). However, data on invertebrate herbivores in these fragile ecosystems remain sparse, despite their pivotal ecological roles.

The grassland caterpillar *Gynaephora alpherakii* offers a valuable model for studying the ecological impacts of climate change in alpine environments. As a prevalent herbivore in the Tibetan meadow ecosystem, this species plays a critical ecological role. During growing seasons, larval densities typically range from several to tens per square meter, with outbreaks reaching up to 200 individuals per square meter (Xi et al. 2013). These caterpillars exhibit temperature‐sensitive feeding behavior, actively foraging during optimal thermal conditions and seeking shelter during extreme heat or cold (Xi et al. 2013). The life cycle and population dynamics of *G. alpherakii* are tightly linked to environmental conditions, making it highly susceptible to climate change (Chen et al. 2015). Research on related species, such as *Gynaephora menyuanensis* and *Gynaephora rossii* , has demonstrated the influence of temperature on herbivore growth, development, and behavior. For instance, warming experiments reveal altered larval development in *G. menyuanensis* (Yu et al. 2016), while studies on *G. rossii* highlight adaptive thermoregulatory behaviors in Arctic environments (Kevan et al. 1982). However, most research focuses on lowland systems, leaving a significant knowledge gap in understanding how high‐elevation herbivores such as *G. alpherakii* respond to climate warming.

To address this, we conducted two complementary experiments examining the effects of experimental warming on the performance of *G. alpherakii*. The first field experiment evaluated feeding behavior, growth, and developmental rates under natural conditions, while the second controlled chamber experiment investigated physiological metrics such as appetite, respiration rate, and body weight changes. We hypothesized that (1) warming would reduce caterpillar body size and (2) behavioral adaptations would not fully mitigate physiologically driven weight loss. This study aimed to provide empirical insights into the impacts of warming on high‐elevation herbivorous insects, contributing to a better understanding of climate change effects on alpine ecosystems.

## 2 Material and Methods

### 2.1 Study Site and Species

This study was conducted in an alpine meadow located in Hongyuan County (32°48′–32°52′ N and 102°01′–102°33′ E), Sichuan Province, China, within the eastern Tibetan Plateau. Detailed information regarding the climatic conditions and plant community composition can be found in prior studies (Wu et al. 2021).

*G. alpherakii* belongs to the genus Spodoptera within the family Lymantriidae (order Lepidoptera) and is recognized as one of the most significant pest species in alpine meadows at elevations ranging from 3200 to 4500 m. We identified the taxonomic classification of *G. alpherakii* based on the comprehensive documentation provided by Yan (2006) and Zhou and Yin (1979), which detail the species' characteristics and retrieval methods in Tibetan alpine meadows (see details in the Appendix S1). This grass moth exhibits a holometabolous life cycle, encompassing four developmental stages: egg, larva, pupa, and adult, with one generation produced annually. Male larvae typically pass through six instars, while female larvae undergo seven. The species overwinters during the first instar larval stage, entering diapause to survive the harsh alpine conditions. Sexual dimorphism is prominent in adult individuals, with males and females displaying distinct morphological traits. Beyond differences in genitalia, significant variations exist in size, coloration, and other physical characteristics. For example, female adults are wingless with less prominent antennae, whereas male adults are winged and possess feathery antennae. This sexual dimorphism is thought to optimize energy utilization by enabling sex‐specific morphological adaptations. Such differentiation facilitates energy allocation toward reproductive growth, thereby enhancing the overall fitness and reproductive success of the population.

### 2.2 Field Experiment

According to numerous studies investigating the effects of climate warming on invertebrates (Liu et al. 2011; Hu et al. 2024), we deployed open‐top chambers (OTCs) to simulate warming. We conducted an artificial warming experiment comprising two treatments: (1) nonwarmed and (2) warmed, with six replicates for each treatment. The field experiment commenced on June 1 and concluded on September 4, 2016.

In late May 2016, 12 cylindrical cages (0.5 m in diameter and height) were systematically deployed in a pasture that was grazed by livestock exclusively during the winter months, maintaining a minimum spacing of 3 m between cages. The vegetation within the study area exhibited homogeneity. Each cage was constructed from a robust steel frame encircled and covered with fine steel mesh (0.1 mm thick, 2 × 2 mm mesh size). Following the initiation of the experiment, the tops of the cages were similarly enclosed with steel mesh, and the bases were embedded 20 cm into the soil to prevent herbivore escape. Additionally, six cages were fitted with transparent plastic film (with sunlight transparency exceeding 95%) to create a warmed microenvironment, while the remaining six served as controls (nonwarmed).

Experimental caterpillars (fourth instar, about 0.02 g per individual) were collected from adjacent areas, ensuring that only healthy, medium‐sized individuals were selected for the study. Ten individuals of *G. alpherakii* were introduced into each replicate, reflecting densities observed during both typical and outbreak years. Prior to the initiation of the experiment, larger herbivores (e.g., grasshoppers) and predators (e.g., *Lycosa* sp.) were removed from the cages to eliminate potential confounding effects.

We measured the fresh body mass of five living caterpillars biweekly, carefully collecting individuals from each cage, weighing them using a portable analytical balance (Sartorius, Germany; precision 0.001 g), and then returning them to their respective cages. Additionally, we monitored cocoon production and alive caterpillar number every three days from the emergence of the first cocoon on August 7 until all caterpillars had cocooned by September 7, measuring the length and width of 18 cocoons to calculate their volume using the elliptical volume formula.

We also evaluated the foraging behavior of *G. alpherakii* following the methodology described by Xi et al. (2013). On sunny days, we recorded the number of feeding individuals—termed “feeding frequency”—on an hourly basis from 08:00 to 18:00 (Beijing time). Two observation sessions were conducted during the first 10 days of the study, when all caterpillars were still alive and active. This approach ensured that measurements of feeding frequency were independent of density fluctuations (Xi et al. 2013).

Temperature was monitored using thermometers (model DS1921G, Maxim Integrated Products, Sunnyvale, California, USA) in three cages for each treatment. Over the experimental duration, the mean daily temperature in the warmed treatments was observed to be 1.8°C higher than in the nonwarmed controls (Cao et al. 2022).

### 2.3 Chamber Experiment

To determine the influence of temperature on the performance of *G. alpherakii*, that is, caterpillar appetite, excrement mass, respiration rate, and body weight, we conducted a complementary chamber experiment three times during the period of the field experiment. The chamber was set as a day/night regime of 14/10 h, respectively, and the humidity was set as 45%. Based on the air temperature during the chamber experiments, the temperature gradient ranged from 6°C (during the first trial), 12°C (during the second trial), or 18°C (during the third trial) to 28°C (in the first and second trials) or 25°C (in the third trial). Each gradient level had a 2°C or 1°C difference, resulting in at least five distinct temperature levels. To ensure the consistency and reliability of the measurements, each level was replicated at least four times. Each replicate was a transparent glass box (20, 21, and 17 cm in length, width, and height, respectively). There were at least 40 boxes in total, each containing 0.5 g of fresh leaves from *Scirpus pumilus* . This plant species was chosen because it is abundant in the study area and serves as a representative plant species for the herbivores under investigation. Five healthy, medium‐sized individuals of caterpillars (caught from the field during each trial) were additionally introduced into each of at least 20 boxes, and the left 20 ones without caterpillars. The leaves were collected from the field and cleaned up indoor and cut down into 5‐cm‐long fragments. The end of leaves was enfolded by cotton and then inserted into 2‐mL centrifuge tube after immersed in water. The chamber experiment lasted for 24 h. We recorded the weight of leaves, the body weight of each caterpillar, and the weight of excrement before and after the experiment. The caterpillar appetite was calculated following the protocols of Waldbauer (1968):

$$\text{Appetite}=W-\left(L-\frac{\mathrm{aw}+\mathrm{bL}}{2}\right)$$

where *W* = weight of leaves before the experiment with caterpillars; *L* = weight of leaves after the experiment with caterpillars; *a* = (weight of leaves before the experiment without caterpillars—weight of leaves after the experiment without caterpillars)/weight of leaves before experiment without caterpillars; *b* = (weight of leaves before the experiment without caterpillars—weight of leaves after the experiment without caterpillars)/weight of leaves after experiment without caterpillars.

In addition, the respiration rate of caterpillars was also measured using a non‐steady‐state and automated soil CO_2_ flux system (LI‐8100, LI‐COR Biosciences, Lincoln, NE, USA) with a survey chamber of 10 cm in diameter (835.2 cm^3^ for chamber volume). The temperature gradient was 4°C from 6°C to 28°C. Before measuring, 10 caterpillars were placed in experiment chamber with enough food for 24 h at each treatment (Song et al. 2008). Each treatment contained three replicates.

### 2.4 Data Analysis

Kolmogorov–Smirnov and Levene's tests were used to check for normality of the distribution and variance homogeneity of the sample residuals, respectively. The cocooning rate was arcsine‐transformed to achieve normality. One‐way analysis of variance (ANOVA) was employed to determine the effects of warming on fresh body mass (per observation day), cocooning rate (per observation day), cocoon volume, and egg production per female moth. Once a significant effect was detected, the difference between treatments was determined using *post hoc* Tukey's tests. A generalized linear model (GLM with Poisson errors) was used to test the effect of warming on the number of feeding caterpillars (per observation hour). In addition, linear or exponential fittings were used to determine the relationship between temperature and caterpillar appetite, excrement mass, respiration rate, or change of caterpillar weight, respectively. A stepwise approach was employed to investigate the significant caterpillar physiological factors influencing caterpillar body size. All the data analyses were conducted in SPSS 22.

## 3 Results

### 3.1 Field Experiment

**FIGURE 1:**
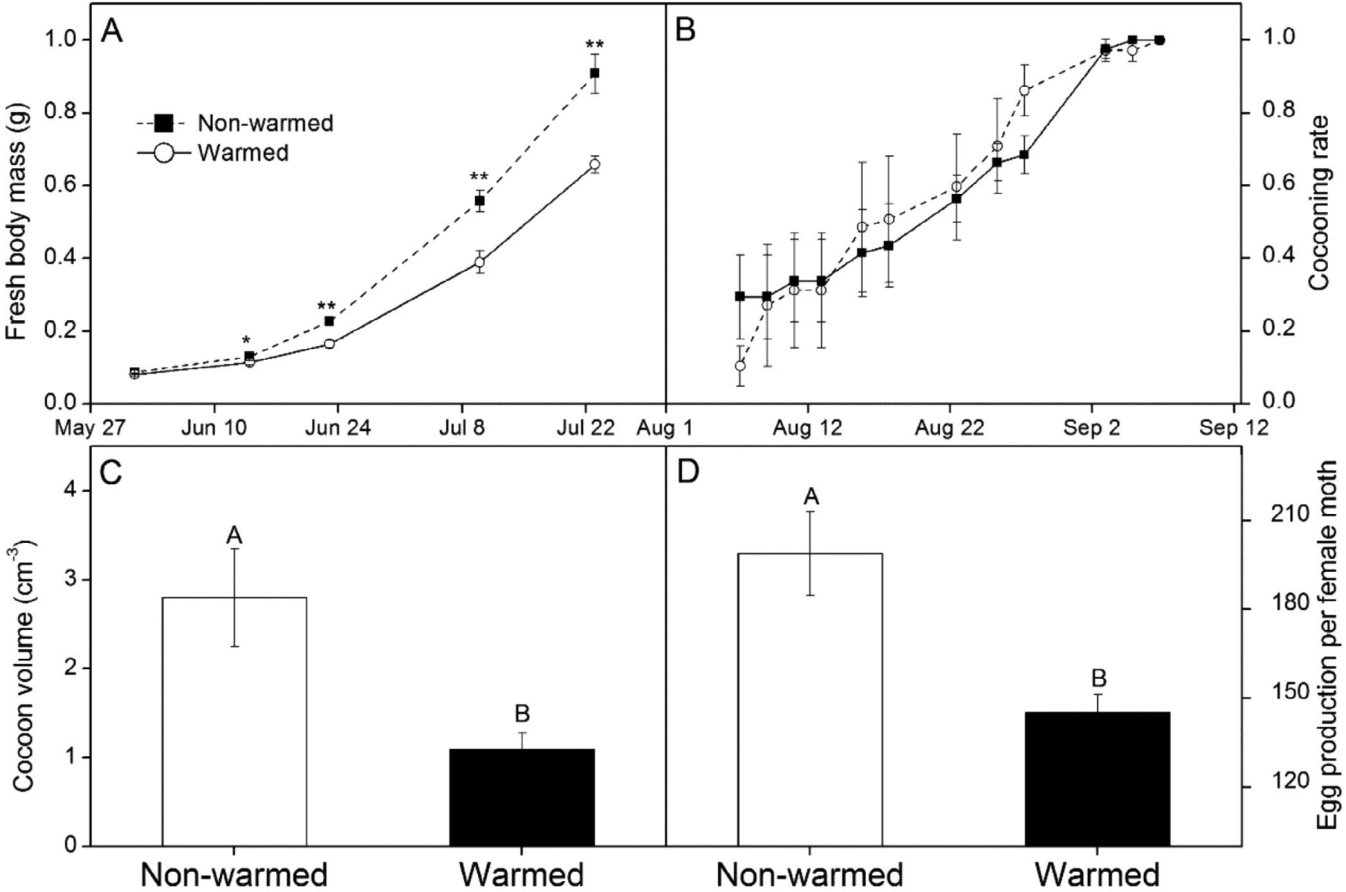
Variations in fresh body mass of caterpillar (A), cocooning rate (B), cocoon volume (C), and egg production per female month (D) among different treatments during the experiment, which are denoted by solid line with hollow circles (nonwarmed) and solid line with solid squares (warmed), respectively. *p < 0.05; **p < 0.01; ***p < 0.001.

Warming significantly decreased fresh body mass of caterpillars by 12.4% in 2014/6/14 (Appendix S1: Table S1: *F* = 7.00, *p* = 0.025), by 27.5% in 2014/6/23 (Appendix S1: Table S1: *F* = 15.69, *p* = 0.003), by 30.0% in 2014/7/10 (Appendix S1: Table S1: *F* = 15.12, *p* = 0.003), by 27.5% in 2014/7/23 (Appendix S1: Table S1: *F* = 18.23, *p* = 0.002) during the observation time (Figure 1A). Warming did not affect cocooning rate (Figure 1B) but significantly decreased cocoon volume by 61.1% (Figure 1C, Appendix S1: Table S1: *F* = 8.59, *p* = 0.015) and egg production per female moth by 26.9% (Figure 1D, Appendix S1: Table S1: *F* = 18.22, *p* = 0.002).

**FIGURE 2:**
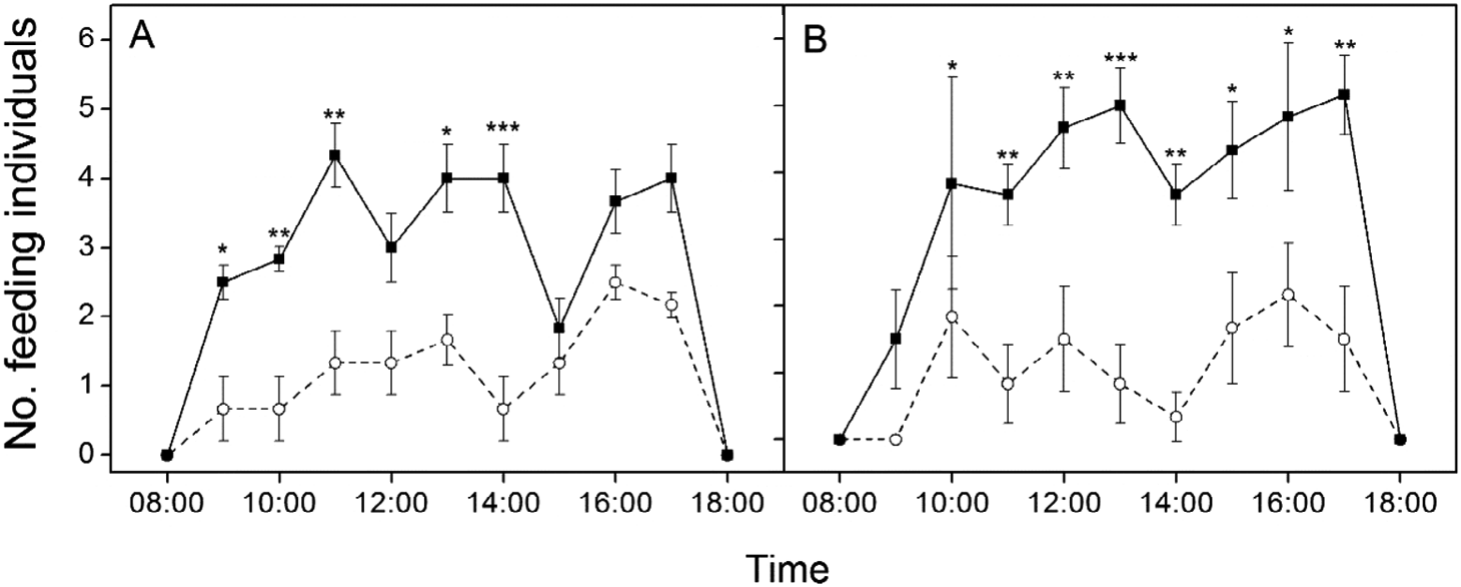
Number of feeding caterpillars (A, the first observation session; B, the second observation session) in the treatments, which are denoted by dotted line with hollow circles or (nonwarmed) and solid line with solid squares (warmed), respectively. *p < 0.05; **p < 0.01; ***p < 0.001.

In addition, warming significantly changed the foraging behavior of caterpillars (Appendix S1: Table S2). Specifically, the number of feeding individuals was significantly higher at 9:00 (Appendix S1: Table S2: *Z* = 2.35, *p* = 0.019), 13:00 (Appendix S1: Table S2: *Z* = 2.33, *p* = 0.020) and 14:00 (Appendix S1: Table S2: *Z* = 3.32, *p* < 0.001) during the first examining time (Figure 2A), and at 10:00 (Appendix S1: Table S2: *Z* = 2.01, *p* = 0.044), 11:00 (Appendix S1: Table S2: *Z* = 2.99, *p* = 0.003), 12:00 (Appendix S1: Table S2: *Z* = 2.96, *p* = 0.003), 13:00 (Appendix S1: Table S2: *Z* = 3.71, *p* < 0.001), 14:00 (Appendix S1: Table S2: *Z* = 3.25, *p* = 0.001), 15:00 (Appendix S1: Table S2: *Z* = 2.57, *p* = 0.010), 16:00 (Appendix S1: Table S2: *Z* = 2.40, *p* = 0.016) and 17:00 (Appendix S1: Table S2: *Z* = 3.27, *p* = 0.001) during the second examining time (Figure 2B) in the warmed plots than in the non‐warmed cages.

### 3.2 Chamber Experiment

**FIGURE 3:**
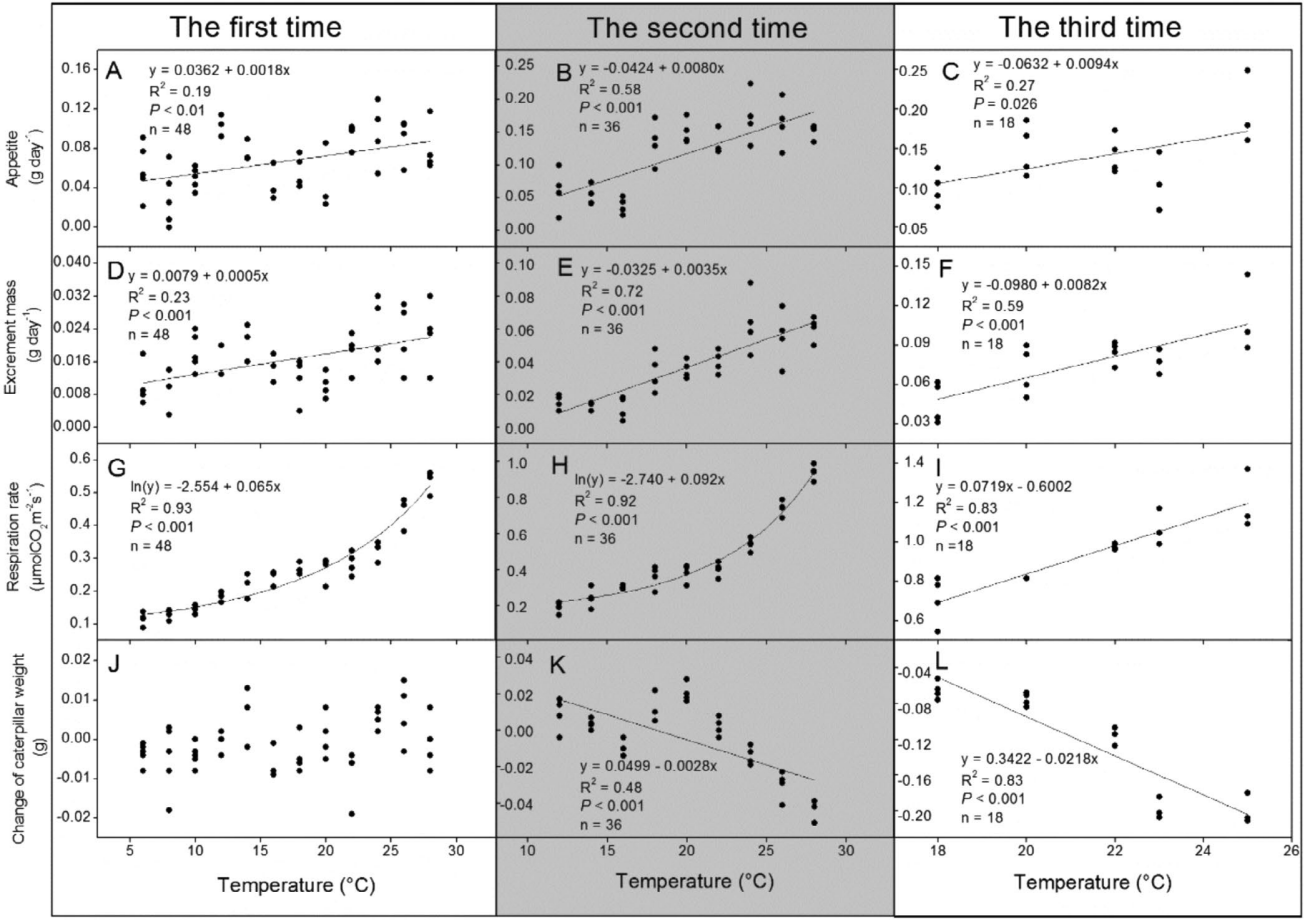
Relationships between temperature and caterpillar appetite (A–C), excrement mass (D–F), respiration rate (G–I), or change of caterpillar weight (J–L) of three examining times in the chamber experiments. Sample size (N), regression coefficients (R^2^), and statistical test (*p* values) are provided for the relationships that are statistically significant.

**FIGURE 4:**
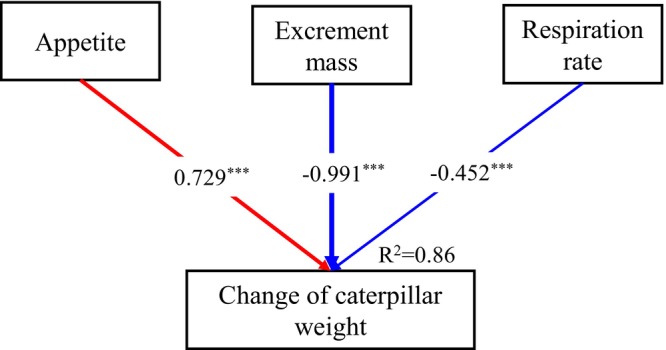
Results of stepwise general linear regression models showing standardization regression coefficients between change of caterpillar weight and caterpillar appetite, excrement mass or respiration rate, respectively. Solid red and blue arrows represent positive/negative paths. The line thickness represents the effect size.*p < 0.05; **p < 0.01; ***p < 0.001.

Caterpillar appetite, excrement mass, and respiration rate increased with temperature in all the three examining times (Figure 3A–I). A negatively linearly relationship was observed between the change of caterpillar weight and temperature in the second (Figure 3K) and third examining times (Figure 3L), except the first examining time (Figure 3J). In addition, results of stepwise regression analysis showed that excrement mass influenced caterpillar weight most (Figure 4, Appendix S1: Table S3: *F* = 203.67, *p* < 0.001, Beta = −0.99).

## 4 Discussion

Our findings demonstrate that artificial warming significantly impacted the performance of the grassland caterpillar *G. alpherakii* in the alpine Tibetan meadow. Elevated temperatures can lead to substantial declines in caterpillar body mass, cocoon volume, and reproductive output. Specifically, the observed reductions of 27.5% in fresh body mass, 61.1% in cocoon volume, and 26.9% in egg production per female moth underscore the potential for warming to adversely affect the population dynamics of this notorious pest species. These present results align with existing literature indicating that climate change can disrupt growth and reproductive strategies across various insect taxa (Desai and Singh 2009; Sheridan and Bickford 2011).

Interestingly, while warming increased feeding time, it did not alter the duration of cocooning. This finding suggests that caterpillars may compensate for the physiological stress induced by higher temperatures through increased feeding efforts (O'Connor 2009). The increase in feeding duration could indicate a behavioral adaptation aimed at counteracting the metabolic stress resulting from warmer conditions (Reuman et al. 2014). However, despite these increased feeding times, the resultant weight loss and decreased reproductive output highlight the inadequacy of this adaptation to fully mitigate the energy deficits associated with elevated temperatures. This conclusion is consistent with previous studies that have shown similar patterns of increased feeding efforts without corresponding increases in body mass or reproductive success under stress conditions (Sahin 2001; Chen et al. 2018; Zhao et al. 2022).

The significant negative correlation between weight change and temperature further supports our hypothesis that warmer conditions have detrimental effects on caterpillar performance. The identification of excrement mass as a major influence on caterpillar weight raises critical questions regarding nutrient assimilation and energy allocation in a warming climate (Ohlberger 2013). Increased metabolic rates may lead to higher energy demands, which, when coupled with potential declines in food quality or availability, could result in reduced growth and fitness (Kingsolver and Huey 1998). Such findings are particularly relevant as they resonate with established theories regarding the temperature‐size rule, where ectothermic organisms tend to exhibit reduced body sizes at elevated temperatures (Atkinson 1994).

From an ecological perspective, the implications of our findings are twofold. First, the decrease in body size and reproductive capacity of *G. alpherakii* could lead to a decline in its population density, potentially altering herbivory dynamics in alpine grasslands. Such shifts could have cascading effects on plant communities, particularly if this species serves as a significant herbivore within the ecosystem. Changes in herbivory patterns may affect plant community structure and dynamics, leading to broader ecological consequences (Post et al. 2009; Cao et al. 2022). Second, the potential for warmer temperatures to shift outbreak patterns of this pest could have significant consequences for grassland management and agricultural practices in the region. Understanding these dynamics is critical for developing effective pest management strategies, especially in the face of ongoing climate change (Li et al. 2020). Additionally, the findings of our study may have implications for the conservation of biodiversity in these high‐elevation ecosystems, where species interactions are already finely balanced (Dong et al. 2023).

While our study offers valuable insights, it also underscores the need for further research to explore the long‐term effects of warming on *G. alpherakii* and other herbivorous species in high elevation areas. Future studies should investigate the interactive effects of multiple stressors, such as changes in precipitation patterns and nutrient availability, to better understand the resilience of these herbivores in a warming world. This approach will provide a more comprehensive view of how climate change affects herbivores and their ecosystems, allowing for better‐informed management decisions.

In conclusion, our findings provide critical evidence of the detrimental effects of climate warming on the performance of grassland caterpillars. This underscores the urgent need for proactive management approaches to mitigate the impacts of climate change on these vital species and their ecosystems. As climate change continues to pose challenges to biodiversity and ecosystem health, understanding the responses of key species like *G. alpherakii* will be essential for ensuring the sustainability of grassland environments.

## Author Contributions

**Rui Cao:** conceptualization (lead), writing – original draft (lead). **Han Chen:** data curation (equal), formal analysis (equal). **Kezhi Zheng:** data curation (equal). **Dajie Nong:** data curation (equal). **Menglei Jiang:** data curation (equal). **Ziyu Zhang:** formal analysis (equal). **Xinwei Wu:** supervision (equal), writing – review and editing (equal). **Peng Xie:** supervision (equal), writing – review and editing (equal).

## Conflicts of Interest

The authors declare no conflicts of interest.

## Supporting information


Appendix S1.

Data S1.

## Data Availability

The authors confirm that the data supporting the findings of this study are available within the article and in Data S1.

## References

[ece370978-bib-0001] Angilletta, M. J. , T. D. Steury , and M. W. Sears . 2004. “Temperature, Growth Rate, and Body Size in Ectotherms: Fitting Pieces of a Life‐History Puzzle.” Integrative and Comparative Biology 44: 498–509. 10.1093/icb/44.6.498. 21676736

[ece370978-bib-0002] Atkinson, D. 1994. “Temperature and Organism Size—A Biological Law for Ectotherms?” Advances in Ecological Research 25: 1–58. 10.1016/S0065-2504(08)60212-3.

[ece370978-bib-0003] Blackburn, T. M. , K. J. Gaston , and N. Loder . 1999. “Geographic Gradients in Body Size: A Clarification of Bergmann's Rule.” Diversity and Distributions 5: 165–174. 10.1111/evo.12726.

[ece370978-bib-0004] Cao, R. , G. Lu , T. Zhang , Z. Li , X. Wu , and S. Sun . 2022. “Invertebrate Herbivory Accelerates Shift Towards Forbs Caused by Warming in a Sedge‐Dominated Alpine Meadow.” Ecosphere 13, no. 9: e4230. 10.1002/ecs2.4230.

[ece370978-bib-0005] Chen, H. , X. Zheng , M. Luo , et al. 2018. “Effect of Short‐Term High‐Temperature Exposure on the Life History Parameters of *Ophraella communa* .” Scientific Reports 8, no. 1: 13969. 10.1038/s41598-018-32262-z. 30228344 PMC6143555

[ece370978-bib-0006] Chen, X. , Y. Liu , and Z. Wang . 2015. “Impact of Climate Change on Gynaephora Alpherakii in Alpine Ecosystems.” Journal of Entomology and Ecology 60, no. 4: 345–356. 10.1016/j.jee.2015.03.004.

[ece370978-bib-0007] Desai, A. S. , and R. K. Singh . 2009. “The Effects of Water Temperature and Ration Size on Growth and Body Composition of Fry of Common Carp, *Cyprinus carpio* .” Journal of Thermal Biology 34: 276–280. 10.1016/j.jtherbio.2009.03.005.

[ece370978-bib-0008] Dong, S. , Y. Zhang , H. Shen , S. Li , and Y. Xu . 2023. “Grassland Biodiversity and Conservation.” In Grasslands on the Third Pole of the World: Structure, Function, Process, and Resilience of Social‐Ecological Systems, 135–172. Springer International Publishing. 10.1007/978-3-031-39485-0_5.

[ece370978-bib-0009] Harvey, J. A. , K. Tougeron , R. Gols , et al. 2023. “Scientists' Warning on Climate Change and Insects.” Ecological Monographs 93, no. 1: e1553. 10.1002/ecm.1553.

[ece370978-bib-0010] Hu, X. , X. Wu , Q. Zhou , et al. 2024. “Warming Causes Contrasting Spider Behavioural Responses by Changing Their Prey Size Spectra.” Nature Climate Change 14, no. 2: 190–197. 10.1038/s41558-023-01918-8.

[ece370978-bib-0011] Hulme, P. E. 1994. “Seedling Herbivory in Grassland: Relative Impact of Vertebrate and Invertebrate Herbivores.” Journal of Ecology 82: 873–880. 10.2307/2261451.

[ece370978-bib-0012] IPCC . 2021. Climate Change 2021: The Physical Science Basis. Contribution of Working Group I to the Sixth Assessment Report of the Intergovernmental Panel on Climate Change. Cambridge University Press. 10.1017/9781009157896.

[ece370978-bib-0013] Kempel, A. , M. Razanajatovo , C. Stein , et al. 2015. “Herbivore Preference Drives Plant Community Composition.” Ecology 96, no. 11: 2923–2934. 10.1890/14-2125.1. 27070012

[ece370978-bib-0014] Kevan, P. G. , T. S. Jensen , and J. D. Shorthouse . 1982. “Body Temperatures and Behavioral Thermoregulation of High Arctic Woolly‐Bear Caterpillars and Pupae (*Gynaephora rossii*, Lymantriidae: Lepidoptera) and the Importance of Sunshine.” Arctic and Alpine Research 14, no. 2: 125–136. 10.1080/00040851.1982.12004289.

[ece370978-bib-0015] Kingsolver, J. , and R. Huey . 2008. “Size, Temperature, and Fitness: Three Rules.” Evolutionary Ecology Research 10, no. 2: 251–268.

[ece370978-bib-0016] Kingsolver, J. G. , and R. B. Huey . 1998. “Evolutionary Analyses of Morphological and Physiological Plasticity in Thermally Variable Environments.” American Zoologist 38, no. 3: 545–560. 10.1093/icb/38.3.545.

[ece370978-bib-0017] Li, S. , H. Zhang , X. Zhou , H. Yu , and W. Li . 2020. “Enhancing Protected Areas for Biodiversity and Ecosystem Services in the Qinghai–Tibet Plateau.” Ecosystem Services 43: 101090. 10.1016/j.ecoser.2020.101090.

[ece370978-bib-0018] Liu, Y. , P. B. Reich , G. Li , and S. Sun . 2011. “Shifting Phenology and Abundance Under Experimental Warming Alters Trophic Relationships and Plant Reproductive Capacity.” Ecology 92, no. 6: 1201–1207. 10.1890/10-2060.1. 21797148

[ece370978-bib-0019] McCary, M. A. , and O. J. Schmitz . 2021. “Invertebrate Functional Traits and Terrestrial Nutrient Cycling: Insights From a Global Meta‐Analysis.” Journal of Animal Ecology 90, no. 7: 1714–1726. 10.1111/1365-2656.13489. 33782983

[ece370978-bib-0020] O'Connor, M. I. 2009. “Warming Strengthens an Herbivore–Plant Interaction.” Ecology 90, no. 2: 388–398. 10.1890/080034.1. 19323223

[ece370978-bib-0021] Ohlberger, J. 2013. “Climate Warming and Ectotherm Body Size–From Individual Physiology to Community Ecology.” Functional Ecology 27, no. 4: 991–1001. 10.1111/1365-2435.12098.

[ece370978-bib-0022] Post, E. , M. C. Forchhammer , M. S. Bret‐Harte , et al. 2009. “Ecological Dynamics Across the Arctic Associated With Recent Climate Change.” Science 325, no. 5946: 1355–1358. 10.1126/science.1173113. 19745143

[ece370978-bib-0023] Reuman, D. C. , R. D. Holt , and G. Yvon‐Durocher . 2014. “A Metabolic Perspective on Competition and Body Size Reductions With Warming.” Journal of Animal Ecology 83, no. 1: 59–69. 10.1111/1365-2656.12064. 23521010

[ece370978-bib-0024] Sahin, T. 2001. “Effect of Water Temperature on Growth of Hatchery Reared Black Sea Turbot, *Scophthalmus maximus* (Linnaeus, 1758).” Turkish Journal of Zoology 25, no. 3: 183–186. https://journals.tubitak.gov.tr/zoology/vol25/iss3/3.

[ece370978-bib-0025] Sheridan, J. A. , and D. Bickford . 2011. “Shrinking Body Size as an Ecological Response to Climate Change.” Nature Climate Change 1, no. 8: 401–406. 10.1038/nclimate1259.

[ece370978-bib-0026] Song, J. , Z. Lu , D. Wang , W. Wu , Y. Su , and J. Wang . 2008. “Respiration and Water‐Loss Characteristics in a Desert Insect *Julodis variolaris* Pallas (Coleoptera: Buprestidae).” Acta Entomologica Sinica 51, no. 2: 132. 10.1016/S1005-9040(08)60003-3.

[ece370978-bib-0027] Waldbauer, G. P. 1968. “The Consumption and Utilization of Food by Insects.” Advances in Insect Physiology 5: 229–288. 10.1016/S0065-2806(08)60230-1.

[ece370978-bib-0028] Wu, X. , J. E. Duffy , P. B. Reich , and S. Sun . 2011. “A Brown‐World Cascade in the Dung Decomposer Food Web of an Alpine Meadow: Effects of Predator Interactions and Warming.” Ecological Monographs 81, no. 2: 313–328. 10.1890/10-0808.1.

[ece370978-bib-0029] Wu, X. , Y. Wang , and S. Sun . 2021. “Long‐Term Fencing Decreases Plant Diversity and Soil Organic Carbon Concentration of the Zoige Alpine Meadows on the Eastern Tibetan Plateau.” Plant and Soil 458: 191–200. 10.1007/s11104-019-04373-7.

[ece370978-bib-0030] Xi, X. , J. N. Griffin , and S. Sun . 2013. “Grasshoppers Amensalistically Suppress Caterpillar Performance and Enhance Plant Biomass in an Alpine Meadow.” Oikos 122, no. 7: 1049–1057. 10.1111/j.1600-0706.2012.00126.x.

[ece370978-bib-0031] Xi, X. , X. Wu , S. Nylin , and S. Sun . 2016. “Body Size Response to Warming: Time of the Season Matters in a Tephritid Fly.” Oikos 125, no. 3: 386–394. 10.1111/oik.02521.

[ece370978-bib-0032] Yan, L. 2006. “Studies of Taxonomy, Geographic Distribution in *Gynaephora* Genus and Life‐History Strategies on *Gynaephora menyuanensis* .” PhD Thesis, Lanzhou University.

[ece370978-bib-0033] Yu, X. C. , K. L. Chen , B. Q. Yao , et al. 2016. “Effects of Simulated Warming on the Growth and Development of *Gynaephora Menyuanensis* Larvae.” Acta Ecologica Sinica 36: 8002–8007.

[ece370978-bib-0034] Zhao, C. , H. Chen , J. Guo , and Z. Zhou . 2022. “Effects of Fluctuating Thermal Regimes on Life History Parameters and Body Size of *Ophraella communa* .” Insects 13, no. 9: 821. 10.3390/insects13090821. 36135522 PMC9504774

[ece370978-bib-0035] Zhao, J. , Y. Yang , X. Xi , and S. Sun . 2014. “Artificial Warming Increases Size at Metamorphosis in Plateau Frogs (*Rana kukunoris*) in the Presence of Predators.” Aquatic Ecology 48: 423–434. 10.1007/s10452-014-9495-y.

[ece370978-bib-0036] Zhou, Y. , and X. Yin . 1979. “A Taxonomic Study on the Steppe Caterpillars (Lepidoptera: Lymantriidae).” Entomotaxonomia 1, no. 1: 23–28.

