# Supplementary material for: Effects of Climate Warming on the Performance of Gynaephora alpherakii (Lepidoptera: Lymantriidae) Larvae in a Tibetan Alpine Meadow
Source: Ecol Evol. 2025 Feb 9;15(2):e70978. doi: 10.1002/ece3.70978 (PMC11807702; doi:10.1002/ece3.70978)
Supplement: Supplementary file 1 — Appendix S1. [file ECE3-15-e70978-s002.docx]

Appendix S1

Effects of climate warming on the performance of Gynaephora alpherakii (Lepidoptera: Lymantriidae) larvae in a Tibetan alpine meadow

Rui Cao, Han Chen, Kezhi Zheng, Dajie Nong, Menglei Jiang, Ziyu Zhang,Xinwei Wu, Peng Xie

**Table S1.** Results of one-way ANOVAs showing the effect of warming on fresh body mass of caterpillars on each monitoring date, on cocoon volume and on egg production per female moth. Degree of freedom (DF), Sum of squares (SS), *F* and *P* values are provided.

| Response variable | Date | Source | DF | SS | *F* | *P* |
| --- | --- | --- | --- | --- | --- | --- |
| Fresh body mass | 2014/6/1 | Warming | 1 | 0.0001 | 0.72 | 0.416 |
|  |  | Error | 10 | 0.0016 |  |  |
|  | 2014/6/14 | Warming | 1 | 0.0008 | 7.00 | **0.025** |
|  |  | Error | 10 | 0.0011 |  |  |
|  | 2014/6/23 | Warming | 1 | 0.0117 | 15.69 | **0.003** |
|  |  | Error | 10 | 0.0075 |  |  |
|  | 2014/7/10 | Warming | 1 | 0.0838 | 15.12 | **0.003** |
|  |  | Error | 10 | 0.0555 |  |  |
|  | 2014/7/23 | Warming | 1 | 0.1875 | 18.23 | **0.002** |
|  |  | Error | 10 | 0.1029 |  |  |
| Cocoon volume | / | Warming | 1 | 8.77 | 8.59 | **0.015** |
|  |  | Error | 10 | 10.20 |  |  |
| Egg production per female moth | / | Warming | 1 | 8610 | 18.22 | **0.002** |
|  |  | Error | 10 | 472 |  |  |

Values considered significant if p<0.05 (bolded in table)

**Table S2.** Results of generalized linear mixed model (with Poisson error) showing the differences in the number of feeding individuals of caterpillars in each observation time. Degree of freedom (DF), Z and *P* values are provided.

| Observation  Time |  | First monitoring | | |  | Second monitoring | | |
| --- | --- | --- | --- | --- | --- | --- | --- | --- |
|  |  | DF | *Z* | *P* |  | DF | *Z* | *P* |
| 8:00 |  | 1,10 | 0.00 | 1.000 |  | 1,10 | 0.00 | 1.000 |
| 9:00 |  | 1,10 | 2.35 | **0.019** |  | 1,10 | 0.01 | 0.996 |
| 10:00 |  | 1,10 | 2.60 | **0.009** |  | 1,10 | 2.01 | **0.044** |
| 11:00 |  | 1,10 | 2.92 | **0.004** |  | 1,10 | 2.99 | **0.003** |
| 12:00 |  | 1,10 | 1.91 | 0.056 |  | 1,10 | 2.96 | **0.003** |
| 13:00 |  | 1,10 | 2.33 | **0.020** |  | 1,10 | 3.71 | **<0.001** |
| 14:00 |  | 1,10 | 3.32 | **<0.001** |  | 1,10 | 3.25 | **0.001** |
| 15:00 |  | 1,10 | 0.69 | 0.493 |  | 1,10 | 2.57 | **0.010** |
| 16:00 |  | 1,10 | 1.14 | 0.253 |  | 1,10 | 2.40 | **0.016** |
| 17:00 |  | 1,10 | 1.78 | 0.075 |  | 1,10 | 3.27 | **0.001** |
| 18:00 |  | 1,10 | 0.00 | 1.000 |  | 1,10 | 0.00 | 1.000 |

Values considered significant if p<0.05 (bolded in table)

**Table S3.** Results of stepwise regression analysis

| Results of stepwise regression analysis (n=102) | | | | | | | |
| --- | --- | --- | --- | --- | --- | --- | --- |
|  | Nonnormalized coefficient | | Standardization coefficient | t | p | Collinearity diagnostics | |
|  | B | SE | Beta |  |  | VIF | Tolerance |
| Constant | 0.003 | 0.005 | - | 0.556 | 0.579 | - | - |
| Excrement mass | -1.928 | 0.211 | -0.991 | -9.122 | **<0.001** | 8.364 | 0.12 |
| Appetite | 0.758 | 0.074 | 0.729 | 10.258 | **<0.001** | 3.577 | 0.28 |
| Respiration rate | -0.077 | 0.013 | -0.452 | -5.765 | **<0.001** | 4.365 | 0.229 |
| R^2^ | 0.862 | | | | | | |
| Adjusted R^2^ | 0.858 | | | | | | |
| F | F (3,98)=203.672,p**<0.001** | | | | | | |
| D-W | 0.901 | | | | | | |
| Dependent variable：Change of caterpillar weight | | | | | | | |
| Values considered significant if p<0.05 (bolded in table) | | | | | | | |

**Gynaephora genus Identification Key (Based on Male Genitalia)**

Cited from Zhou & Yin (1979)

1. The length of the clasping organ is 1.5 times its height, slightly rectangular, with the dorsal and ventral edges parallel and curved upwards, and the tip pointed... ***Gynaephora alpherakii***
   1'. The length of the clasping organ is not more than its height, not rectangular, with the dorsal and ventral edges not curved upwards, and the tip not pointed... 2
2. The clasping organ is slightly square, with the dorsal and ventral edges of similar length... 3
   2'. The clasping organ is slightly trapezoidal, with the dorsal edge significantly longer than the ventral edge... 4
3. The clasping organ is rhomboid, with a blunt projection at the tip; the penis is U-shaped, with the basal and distal halves of equal length... ***G.*** ***qinghaiensis***
   3'. The clasping organ is round or square, with no projection at the tip; the penis has a slight curve, with the distal half longer than the basal half... ***G.*** ***minora***
4. The clasping organ has equal length and height, with the tip bluntly protruding backwards, and the penis straight... ***G.*** ***rouergensis***
   4'. The clasping organ is shorter than its height, with a rounded blunt tip, and the penis is curved... ***G.*** ***aureate***

Zhou, Y., & Yin, X. (1979). A taxonomic study on the steppe caterpillars (Lepidoptera: Lymantriidae). *Entomotaxonomia*, 1(1), 23-28.
